# Multiaxial Deformations of Elastomeric Skins for Morphing Wing Applications: Theoretical Modeling and Experimental Investigations

**DOI:** 10.3390/polym14224891

**Published:** 2022-11-12

**Authors:** Dilshad Ahmad, Deepak Kumar, Rafic M. Ajaj

**Affiliations:** 1 Department of Aerospace Engineering, Khalifa University of Science and Technology, Abu Dhabi 127788, United Arab Emirates; 2 Department of Mechanical Engineering, Maulana Azad National Institute of Technology Bhopal, Bhopal 462003, India

**Keywords:** elastomers, hyperelasticity, multiaxial modeling, morphing wing, flexible skin

## Abstract

An elastomeric class of flexible skin-based polymorphing wings changes its configuration to maximize performance at radically different flight conditions. One of the key design challenges for such an aircraft technology is the multiaxial deformation characterization and modeling of nonlinear elastomeric skins of polymorphing wings. In the current study, three elastomeric materials, Latex, Oppo, and Ecoflex, are experimentally characterized and modeled under all possible deformation modes such as uniaxial, pure shear, biaxial, and equibiaxial relevant for flexible skin-based morphing wing applications. Additionally, a novel material model with four material constants is proposed to model the considered elastomers-based morphing wings keeping all the material parameters constant for all the possible deformation modes. The present experimental and theoretical study provides a concise comparative study of the three elastomers used in the morphing wings tested in all possible deformation modes.

## 1. Introduction

In a traditional aircraft, only one or two flight conditions are optimized rather than optimizing the entire flight envelope. At the same time, the advancement of research is moving closer to nature, mimicking the flight of birds. In particular, birds reshape their wing and adjust their profiles to obtain optimal performance in all flight conditions [1,2,3,4]. Recently, morphing wings offer excellent aerodynamic efficiency and control authority for an aircraft over a wide range of flight conditions. Moreover, the morphing wing provides a potential solution to the rigid aircraft wings with hinged ailerons or flaps connections that account for noise and vibration in the airframe [5,6,7]. To this end, lightweight, flexible elastomeric materials are implemented on the ribs and morphing structures of the wing. Recently, the focus of the morphing wing has been shifted from a monomorphing wing (one degree of freedom) to a polymorphing wing (two or more degrees of freedom). Hence, elastomers are a potential candidate material for morphing skin due to their multiaxial seamless deformation, low in a plane, and high out-of-plane stiffness with reduced actuation force requirement [8,9].

Researchers have implemented elastomers in the morphing wing, and their mechanical characterization, simulation, and material modeling are investigated. For this purpose, they either purchased different elastomers or prepared them in the laboratory before implementing them on the morphing structures of the wing. Kikuta [10] is a pioneer in the field who investigated different elastomers in the pursuit of ideal skin material for morphing wing applications. They investigated Tecoflex (80A, 93A, and 100A), Ritiflex (663A and 640A), Arnitel, and Shape memory polymer. Mainly they have conducted uniaxial and equibiaxial experiments and concluded that TEcoflex 80A would be the best candidate material for the morphing applications. Thill et al. [11] presented a comprehensive review of the candidate materials for morphing skins. They demonstrated that elastomeric skins have low crosslink density and are useful for polymorphing where multiaxial high strains are required. Peel et al. [12] fabricated a morphing skin by reinforcing carbon fiber in polyurethane elastomers to bear a more aerodynamic load. Their experimental and simulation results showed smooth elastic cambering and no buckling or waviness in the skins. Bishay and Aguilar [13] proposed a hybrid morphing skin with composites and elastomers aligned periodically. Their computational parametric analysis showed that torsional compliance could be increased by increasing the width ratio and decreasing the number of elastomeric sections and elastomers’ torsional rigidity. Bubert et al. [14] fabricated an elastomer-based skin that strained smoothly to 100% global strain with 100% area change. Finite element simulations were conducted to achieve 30% global strain with 1.5% maximum local strain. Ajaj et al. [15] developed a zigzag wing box-based morphing wing utilizing rigid and flexible parts together. Latex-based elastomeric skin is implemented as a flexible part. Their complete mathematical analysis showed that a maximum of 44% span extension of the wing is achieved due to the integration of the elastomeric part. In a series of works, Olympio and Gandhi [16,17,18] conducted comprehensive experimental and theoretical studies on various flexible elastomer-based morphing wings. Their results highlighted the advantages of implementing elastomeric skins on different types of cores of a morphing wing. Woods and Friswell [19] developed an elastomer-based skin for morphing wing application termed Adaptive Aspect Ratio (AdAR) wing. They tested the silicone-based elastomeric skin under the uniaxial mode of deformation, and analytical optimization of the skin was done. Further, Woods and Heeb [20] fabricated a unique, TPU-based morphing skin with the help of a multi-nozzle 3D printer. They named it Geometrically Anisotropic ThermOplastic Rubber (GATOR), and a detailed analytical optimization study proved that the skin is better suited for low in-plane and high out-of-plane stiffness. In another work from the same group, Rivero et al. [21] designed a modular FishBAC wing with 3D-printed skins. Uniaxial stress-strain curves are plotted to understand the nonlinear mechanical behavior of the skin. Parancheerivilvilakkath et al. [22] developed a Latex-based polymorphing wing capable of chord and camber morphing. The design, modeling, and mechanical testing of the Latex-made wing achieved 10% chord extensions and 20% camber changes.

The above literature studies show that flexible elastomers are currently used in morphing wings along with rigid structures such as ribs. At the same time, the hyperelastic material modeling under multiaxial deformation modes is of utmost importance for simulation and modeling the entire wing structure. On the contrary, modeling such hyperelastic materials is generally done using only one mode of deformation at one time. For example, the uniaxial mode of deformation is selected to understand the hyperelastic behavior of elastomers through different hyperelastic mathematical models [23,24,25,26]. Moreover, a few researchers investigated the influence of three deformation modes (uniaxial, pure shear, and equibiaxial) on various constitutive models [27,28,29,30,31]. Besides the developments of different material models for the hyperelastic response of elastomeric material class, choosing an appropriate model for a specific application, for example, the morphing wing, which often deforms in biaxial mode (in the polymorphing wings), is still challenging [32,33,34]. The central aim of such phenomenological defined model expressions of polynomial, exponential, and logarithmic terms [35,36,37] is to capture the experimental data accurately, in particular, a uniaxial test of deformation. At the same time, the fitting accuracy of other deformation modes such as pure shear, biaxial, and equibiaxial tests were majorly ignored in capturing the material response of elastomers with the same set of material constants fitted with uniaxial test data. To the best of the authors’ knowledge, most of the researchers compared uniaxial, pure shear, and equibiaxial experiments to propose different material models. However, the biaxial deformation mode (unequal strain rate in the X and Y direction) is not investigated in detail. Only Ahmad et al. [38] carried out a biaxial deformation test experimentally and compared it with other modes of deformations such as uniaxial, pure shear, and equibaixial only for Latex.

Motivated by the literature, the current research aims to characterize three categories of elastomeric materials experimentally, Latex (widely applicable), Oppo (highly durable), and Ecoflex (less viscous) used in aircraft morphing wings by developing a novel material model keeping all the material parameters constant for all the possible modes of deformations. The present study is further organized as follows: Section 2 discusses all the experimental details of the mechanical characterization of three different elastomeric materials. Section 3 revisits the material modeling of an incompressible isotropic hyperelastic elastomeric material class of skins for morphing wing applications subjected to different modes of deformations. Furthermore, a novel material model with four material constants is proposed to model the considered elastomers-based morphing wings keeping all the material parameters constant throughout all the possible modes of deformations in the same Section 3. Later, Section 4 validates the analytical findings of a newly proposed material model in previous Section 3 and identifies the material constants of the model for each experimentally tested Latex, Oppo, and Ecoflex elastomers. Section 4 also discusses a summarized mechanical comparison of the elastomers used in morphing wing applications, connecting with the experimentally validated analytical findings of the current study. At last, Section 5 summarizes the conclusions drawn from the present work in the context of morphing wings.

## 2. Experimental

In the present section, details of the multiaxial testing set up to conduct the experiments such as uniaxial (UX), pure shear (PS), biaxial (BX) and equibiaxial (EB) at a particular condition is elaborated. Moreover, the geometry of the specimen and the fabrication method of Ecoflex are discussed in a separate section.

### 2.1. Experimental Set Up

**Figure 1:**
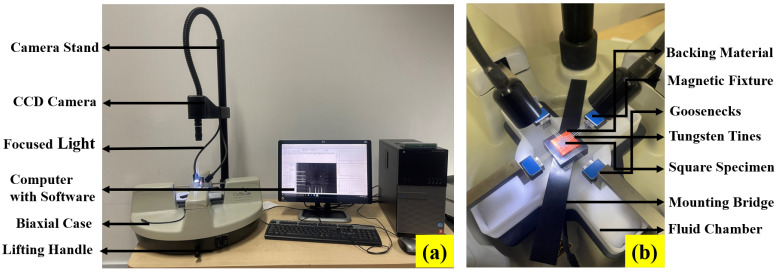
Representation of (**a**) Biaxial testing device and (**b**) enlarged view of the specimen holder for conducting uniaxial, pure shear, biaxial and equibiaxial test at a fixed strain rate [40].

The experimental setup to conduct the multiaxial test on the three elastomers Latex, Oppo and Ecoflex is shown in Figure 1. The device is compact and easy to use, and it is called Biaxial Planar Tensile Testing Device (Make: CellScale, Waterloo). All the tests such as UX, PS, BX, and EB are conducted at a comparatively slow deformation strain rate to assess the long-term behavior of the elastomers. The elastomer-based skins are generally used in morphing wings of Unmanned Aerial Vehicles (UAVs) flying at low altitudes and speeds [11]. At this low altitude and speed, environmental factors such as temperature, humidity, and ambient pressure do not have a significant role to play in elastomeric materials. Hence, all the materials are tested at room temperature for a proposed modeling perspective. The maximum strain achieved during the test is 100%. As shown in Figure 1, the biaxial device consists of a compact biaxial test setup integrated with LabJoy software. The LabJoy software is used to analyze the data and process the image to obtain strain maps. Figure 1b shows the enlarged view of the testing arrangement. The device consists of four actuators fixed at four sides of the device through goose-necks. Four magnet-attached grippers are situated at the four corners connected with each actuator individually. Each gripper has five tungsten-made tiny tines pierced into the specimen to hold it. The diameter and depth of each tine are 305 μm and 1.9 mm, respectively, which adequately grips the specimen. The square-shaped specimen is fixed in the gripper with the help of a mounting bridge that moves up and down with the help of a fluid chamber, as shown in Figure 1b. A CCD camera is fixed at the top of the specimen through a camera stand, as shown in Figure 1a. This camera provides high-resolution video of the specimen during the experiments that are further analyzed in the LabJoy software. Strains are calculated through images in the software. To this end, a square is created on the first image of the specimen, and henceforth strains are created in the region as the deformation takes place, similar to Helal et al. [39]. The highest capacity of the load cell is 5N which provides the force needed to extend the specimen under different modes of deformations discussed in the forthcoming Section 2.2. Engineering stress is then calculated by dividing the force value by the original cross-sectional area of the specimen. The original area is obtained by dividing the width by the thickness of the specimen. All the tests are conducted at least five times to ensure repeatability of the test. For representation purposes, one experimental data point is used to fit the data in each condition as detailed in Section 3.

### 2.2. Specimen Geometry, Elastomer Synthesis, and Experimental Conditions

**Figure 2:**
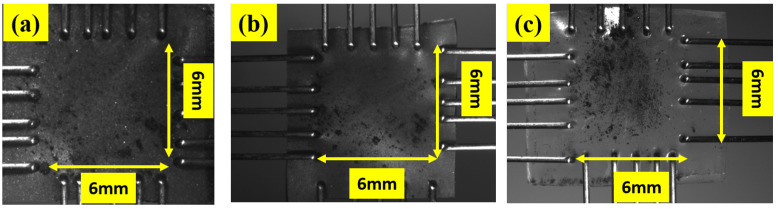
Dimension of specimens used for biaxial testing of three different elastomers (**a**) Latex (**b**) Oppo and (**c**) Ecoflex [40].

The specimens of Latex, Oppo, and Ecoflex are cut into a square shape of side 7 mm using a scissor. Grips are fixed from all four sides keeping the end-to-end distance of 6 mm as shown in Figure 2. We have tested the elastomers of square shape of side 6 mm because of the limitation of the Biaxial Machine [39]. However, increasing the specimen size does not significantly affect the parameters of the elastomers, as experimentally verified by Pharr et al. [41]. Graphite powder is sprinkled over the specimen before each test starts, so strain maps are easily obtained. Among the three elastomers selected, Latex and Oppo are from the natural rubber class, while Ecoflex is from the silicone family. These are commercially available low-cost elastomers. Latex is the most widely used elastomer in the morphing wing and is directly purchased from Radical Rubber (www.radicalrubber.co.uk, accessed on 10 March 2022), while Oppo is purchased from Oppo Medical Inc., Seattle, WA, USA. The thickness of both sheets is 0.25 mm. Latex and Oppo exhibit high-quality finish with translucent and blue colors, respectively. Oppo is selected as it is already used for medical purposes in physiotherapy. Therefore, its durability is already proven and can undergo multiple cycles without failure [42]. Ecoflex is purchased from Smooth-ON, USA, and this is selected because silicone-based elastomers are being used in morphing wings. Silicone-based Ecoflex elastomer is synthesized in the laboratory by mixing two parts (Part A and Part B) in equal proportion by weight. Then the mixture is adequately mixed with a stirrer for 3–5 min. This is spread in a 0.5 mm thick mold made of acrylic sheet. The whole mixture is then evenly settled in the mold using a hand-made applicator. The mold filled with the mixture is then kept open at room temperature to dry for 4 h. The sheet is then ready to use. To confirm its thickness, the thickness of the sheet is measured with the help of a portable thickness gauge (Model: Yunir1z5xbr97ut, Make: Yunir). The thickness is measured from at least ten different places of the fabricated sheet to confirm the thickness. The thickness was then found to be in the range of 50 mm ± 0.04 μm.

### 2.3. Various Mechanical Tests under Multiaxial Modes of Deformation

**Figure 3:**
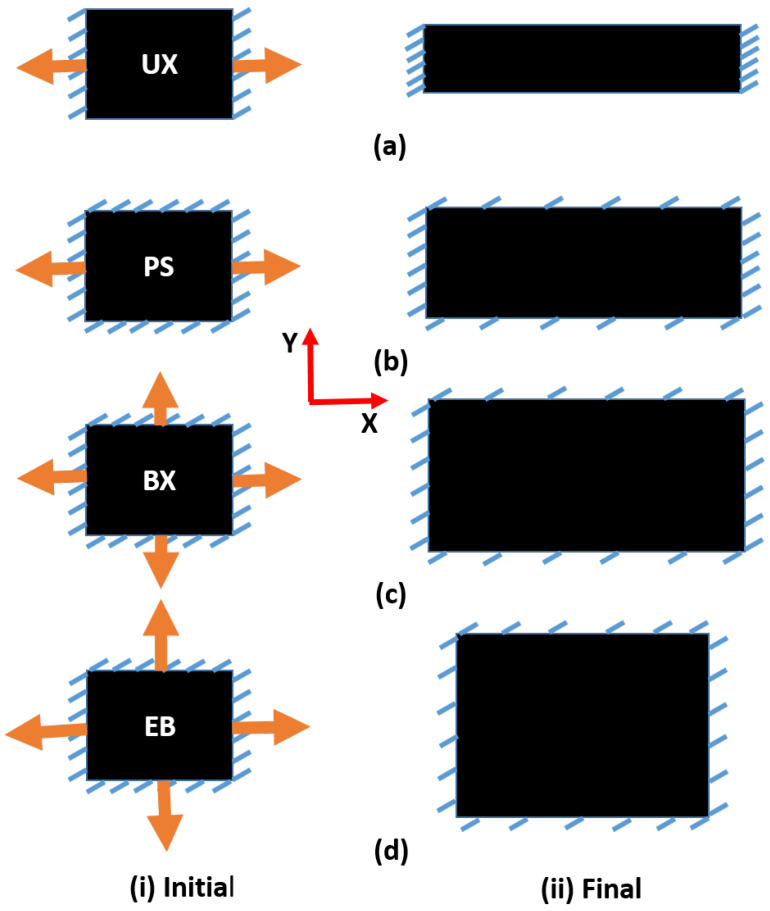
Representation of multi axial deformation modes at the (**i**) initial and (**ii**) final position of the specimen. The various deformation modes are shown in (**a**) UX (**b**) PS (**c**) BX and (**d**) EB.

Different modes of deformations are elaborated in Figure 3a for uniaxial, Figure 3b for pure shear, Figure 3c for biaxial and Figure 3d for equibiaxial. The initial and final positions of the specimen are elaborated in Figure 3(i) and Figure 3(ii), respectively. In the uniaxial mode of deformation, as shown in Figure 3a, the square-sized specimen is fixed from the loading side (X direction) and the transverse side (Y direction) is free to contract. At the same time, the load is applied from the X direction. In this way, the specimen deforms continually from the Y direction during the testing. In the pure shear mode of deformation, the specimen is fixed from all sides, and loading is applied from the X direction keeping the Y direction fixed to prevent the contraction of the specimen as shown in Figure 3b. For the biaxial deformation mode, loads are applied from both X and Y directions, but loading from the Y direction is slower than that of the X direction, as shown in Figure 3c. This is an example of unequal loading from lateral and transverse directions. For the equibiaxial mode of deformation, equal loads are applied from both the lateral and transverse directions, as shown in Figure 3d.

### 2.4. Strain Measurement under Various Modes of Deformations

**Figure 4:**
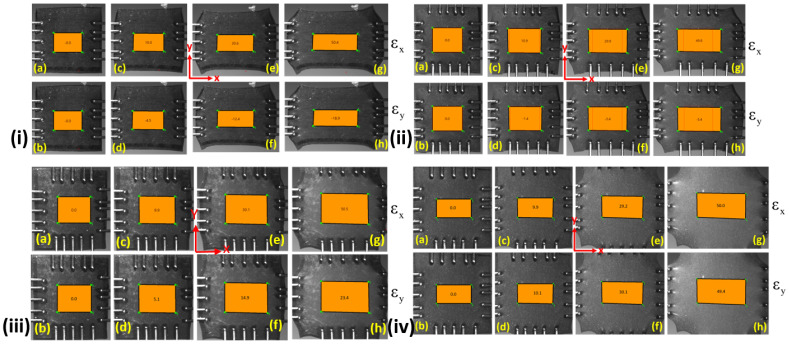
Strain maps developed under all possible modes of deformation are represented for (i) UX (ii) PS (iii) BX (iv) EB in the biaxial testing device. The strain maps for X direction are shown in (**a**,**c**,**e**,**g**) and corresponding strain maps for Y direction are shown in (**b**,**d**,**f**,**h**), respectively for each deformation modes.

The CCD camera, as shown in Figure 1 is used to measure the real-time deformation of the specimen. The bi-axial machine has an integrated image analysis software called ’Labjoy’ that captures images to provide strains while testing through the DIC technique. The analysis can be done by selecting the first image and making a square shape in the middle of the specimen. The source and target images are first preset, and then all the square region points are tracked. This will create strains in the middle of the square region for all the images and can be visualized after selecting. This way, strains in the X and Y directions can easily be visualized. All the strains obtained for various deformation modes are elaborated in Figure 4. Strains in the X and Y directions are $\varepsilon_{x}$ and $\varepsilon_{y}$, respectively, as shown in Figure 4i. Under the uniaxial mode of deformation, the specimens at 0% strain in both the X and Y directions are represented in Figure 4i(a,b). The strain maps developed in the X direction are 10%, 30.6%, and 50.4%. Their corresponding strain rates in the Y direction are −4.5%, −12.4%, and −18.9%, respectively as shown in Figure 4i(a), (b), (c), (d), (e), (f), (g), and (h), respectively. Here, $\varepsilon_{y}$ at different positions are negative, showing the specimen contraction in the Y direction. Hence, an overall contraction of 40% is observed in the transverse direction when the specimen is fully stretched under the uniaxial mode of deformation.

Under the pure shear mode of deformation, the strains developed in the X and Y direction at different specimen positions are shown in Figure 4ii. The initial strain maps are 0% in the X and Y directions, as shown in Figure 4ii(a,b). It is clearly shown that as the strain in the X direction reaches 50%, the corresponding strain in the Y direction is around −5%. This kind of loading is termed pure shear loading when the transverse contraction (Y) is less than 10% while extension takes place in the lateral direction (X) [30,43]. They enable the pure shear mode of deformation test of elastomers in their works by taking a very wide sample and keeping the width to height ratio greater than 10. This arrangement keeps the lateral contraction of the specimen within the allowable limit of −10%. In the current work, the lateral contraction is prevented using a unique gripping system of the biaxial machine. It consists of tungsten-made small flexible tines which are pierced in the specimen. These flexible tines move apart easily from the transverse direction when extension occurs from the lateral direction. This way, the lateral contraction is shown to be around −5%. as shown in Figure 4ii(h). Furthermore, it is clearly shown from Figure 4ii that strains developed are $\varepsilon_{x}=0\%,10.9\%,29.9\%$ and 49.8% in the X direction and their corresponding strains in the Y direction are $\varepsilon_{y}=0\%,-1.4\%,-3.4\%$ and −5.4%, respectively. A negative sign indicates that the contraction takes place in the Y direction. Hence, the strains for pure shear deformation at different positions in the Y direction are always under 5.4%.

The strain maps for both the X and Y directions under biaxial deformation modes are shown in Figure 4iv. In this mode, the extension in the Y direction is considered lesser than that of the X direction at a particular time, as shown in Figure 4iii(a–h). Hence, $\varepsilon_{x}=0\%,9.9\%,30.1\%$ and 50.5% and the corresponding strains in the Y direction are $\varepsilon_{y}=0\%,5.1\%,14.9\%$ and 23.4%, respectively. Furthermore, the strain maps for equibiaxial mode of deformation Figure 4iv(a–h). In the equibiaxial mode of deformation, the loading rate in both the X and Y directions is the same. Therefore, strain maps observe in both directions are the same. For example, the strains along the X direction are $\varepsilon_{x}=0\%,9.9\%,29.2\%$ and 50.0% and the strains in the Y direction are $\varepsilon_{y}=0\%,10.1\%,30.1\%$ and 49.4% respectively as shown in Figure 4iv.

## 3. Material Modeling

This section summarizes the material modeling of an incompressible isotropic hyperelastic elastomeric material class of skins for morphing wing applications subjected to different modes of deformations by defining the state variables in line with the literature [44,45,46].

### 3.1. Kinematics of Hyperelastic Deformation

Consider a body $\Omega =[P_{k}]$ containing a set of material points $P_{k}$. A reference frame $\phi =[O,e_{i}]$ is set in such a way that it contains the origin *O* and an orthonormal vector space $e_{i}$ in a three-dimensional Euclidean space. The time locus of a position vector X(P,t) relative to $\phi =[O,e_{i}]$ describes the mechanical motion relative to the reference frame ϕ. The material deformation of an incompressible isotropic hyperelastic body is governed by a nonlinear deformation field map k(X,t) that transforms a material point $X\in P_{k}$ onto a current/Eulerian configuration of the material point $x=k_{t}\left(X\right)$. If $T_{X}\Omega_{0}$ and $T_{x}\Omega$ are the tangent spaces in the reference/Lagrangian and current configurations, then the deformation gradient tensor F that maps the unit tangent of the reference configuration onto the current configuration is given by

(1) $$\begin{array}{c} \begin{array}{c} F:T_{X}\Omega_{0}⟶T_{x}\Omega ,\;F=\frac{dx}{dX}. \end{array} \end{array}$$

Further, if dA and dV denote the infinitesimal area and volume elements in the reference configuration, then cof[F] and J=detF characterize the deformed area and volume elements in the current configuration given as

(2) $$\begin{array}{c} \begin{array}{c} \mathrm{cof}\left[F\right]=\frac{da}{dA},\;J=\frac{dv}{dV}. \end{array} \end{array}$$

Let $\left|dx\right|=ds$ and $\left|dX\right|=dS$, where *s* and *S* denote the arc length parameters. Then, the above Equation (1) may be written as

(3) $$\begin{array}{c} \begin{array}{c} \lambda e=FE, \end{array} \end{array}$$

where e=dx/ds and E=dX/dX are unit vectors tangent to the arc length parameters, respectively and λ=ds/dS is represented as a stretch parameter defined as the ratio of the deformed length to the undeformed length of the material element. In the above relation (3), F rotates E in the direction e and stretches it by 0<λ<∞. Physically, it becomes essential to use the polar decomposition theorem [47,48] of linear algebra applied to the nonsingular tensor F as

(4) $$\begin{array}{c} \begin{array}{c} F=RU=VR, \end{array} \end{array}$$

where R denotes the local rigid body rotation of a material element and U, V are the positive and symmetric tensors describing the local deformation of the element and generally known as the right and left stretch tensors, respectively. Physically, the above (4) decomposition of the gradient tensor F is unique and the direct use of the stretch tensors U or V are tedious. Thus, it is customary to use their squares as

(5) $$\begin{array}{c} \begin{array}{c} C=F^{T}F=U^{2},\;B=FF^{T}=V^{2}, \end{array} \end{array}$$

where B and C represents the right and left Cauchy-Green deformation tensors, respectively.

### 3.2. Governing Equations of Hyperelastic Deformation

In general, the energy stored in an isotropic material class during deformation is governed by principal stretches or invariants [44,45,46]. In this context, one may decompose the left Cauchy-Green deformation tensor B and its cof[B] tensor for the corresponding eigenvalues and eigenvectors as

(6) $$\begin{array}{c} \begin{array}{c} B=\sum_{i=1}^{3}\lambda_{i}^{2}N^{i}⊗N^{i},\;\mathrm{cof}\;\left[B\right]=\sum_{i=1}^{3}a_{i}^{2}\lambda_{i}^{2}N^{i}⊗N^{i}, \end{array} \end{array}$$

where $a_{i}=J/\lambda_{i}$ ($a_{1}=\lambda_{2}\lambda_{3}$, $a_{2}=\lambda_{3}\lambda_{1}$ and $a_{3}=\lambda_{1}\lambda_{2}$) denote the principal areal stretches. Thus, the set of principal invariants corresponding to an incompressible isotropic hyperelastic material deformation are given by [36,49]

(7) $$\begin{array}{c} \begin{array}{c} I_{1}=\mathrm{tr}B=\lambda_{1}^{2}+\lambda_{2}^{2}+\lambda_{2}^{2},\\ I_{2}=\mathrm{tr}\left(\mathrm{cof}\;[B]\right)=\frac{1}{2}\left[{\left(\mathrm{tr}B\right)}^{2}-\mathrm{tr}B^{2}\right]=a_{1}^{2}+a_{2}^{2}+a_{3}^{2}=\frac{1}{\lambda_{1}^{2}}+\frac{1}{\lambda_{2}^{2}}+\frac{1}{\lambda_{3}^{2}},\\ I_{3}=J^{2}=\det\;B={\left(\lambda_{1}\lambda_{2}\lambda_{3}\right)}^{2}=1. \end{array} \end{array}$$

From the theory of hyperelasticity [49,50], the Cauchy stress tensor σ for a given invariant-based strain energy density function $W\left(I_{1},I_{2},I_{3}\right)$ is expressed as

(8) $$\begin{array}{c} \begin{array}{c} \sigma =\frac{2}{\sqrt{I_{3}}}B\frac{\partial W(I_{1},I_{2},I_{3})}{\partial B}=\frac{2}{\sqrt{I_{3}}}\left(\frac{\partial W}{\partial I_{1}}\frac{\partial I_{1}}{\partial B}+\frac{\partial W}{\partial I_{2}}\frac{\partial I_{2}}{\partial B}+\frac{\partial W}{\partial I_{3}}\frac{\partial I_{3}}{\partial B}\right). \end{array} \end{array}$$

The derivatives of the invariants (8) with respect to the left Cauchy green deformation tensor B are given by

(9) $$\begin{array}{c} \begin{array}{c} \frac{\partial I_{1}}{\partial B}=I,\;\frac{\partial I_{2}}{\partial B}=I_{1}I-B,\;\frac{\partial I_{3}}{\partial B}=I_{2}I-I_{1}B+B^{2}, \end{array} \end{array}$$

where I is the identity tensor. On using the above relations (8) and (9) for an incompressible balloon actuator made of hyperelastic material, we obtain

(10) $$\begin{array}{c} \begin{array}{c} \sigma =-pI+2\left(\frac{\partial W}{\partial I_{1}}+I_{1}\frac{\partial W}{\partial I_{2}}\right)B-2\left(\frac{\partial W}{\partial I_{2}}\right)B^{2}, \end{array} \end{array}$$

where *p* denotes the indeterminate pressure to be determined from boundary conditions. In the current study, experiments are carried out in a displacement-driven setup wherein the nominal stresses are readily available due to the experimental lack of actual cross-section. In line with that, the above expression (10) is utilized to derive the nominal stress expressions for different modes of deformations such as uniaxial, pure shear, biaxial, and equibiaxial deformations cases.

#### 3.2.1. Uniaxial Mode of Deformation

In this deformation case, the material is stretched in x-direction $\left(\sigma_{11}\neq 0\right)$ while others directions are stress-free $\left(\sigma_{22}=\sigma_{33}=0\right)$. The deformation gradient tensor F and the nominal stress tensor under uniaxial tension applied in the x-direction are given by

(11) $$\begin{array}{c} \begin{array}{c} F=\left[\begin{array}{ccc} \lambda & 0 & 0\\ 0 & 1/\sqrt{\lambda } & 0\\ 0 & 0 & 1/\sqrt{\lambda } \end{array}\right],\;\sigma =\left[\begin{array}{ccc} \sigma_{11} & 0 & 0\\ 0 & 0 & 0\\ 0 & 0 & 0 \end{array}\right]. \end{array} \end{array}$$

Using the above expressions (11) in (10), the nominal stress under uniaxial tension applied in x-direction is obtained as

(12) $$\begin{array}{c} \begin{array}{c} \sigma_{11}=2\left(\frac{\partial W}{\partial I_{1}}+I_{1}\frac{\partial W}{\partial I_{2}}\right)\left(\lambda -\frac{1}{\lambda^{2}}\right)-2\left(\frac{\partial W}{\partial I_{2}}\right)\left(\lambda^{3}-\frac{1}{\lambda^{3}}\right). \end{array} \end{array}$$

#### 3.2.2. Pure Shear Mode of Deformation

In this deformation case, the material is again stretched in the x-direction $\left(\sigma_{11}>0\right)$ while the other directions are constrained $(\sigma_{22}>0$, $\lambda_{2}=1)$ and stress-free $\left(\sigma_{33}=0\right)$. The deformation gradient tensor F and the nominal stress tensor for the given deformation case are given by

(13) $$\begin{array}{c} \begin{array}{c} F=\left[\begin{array}{ccc} \lambda & 0 & 0\\ 0 & 1 & 0\\ 0 & 0 & 1/\lambda \end{array}\right],\;\sigma =\left[\begin{array}{ccc} \sigma_{11} & 0 & 0\\ 0 & \sigma_{22} & 0\\ 0 & 0 & 0 \end{array}\right]. \end{array} \end{array}$$

Using the above expressions (13) in (10), the nominal stress under pure shear deformation case is obtained as

(14) $$\begin{array}{c} \begin{array}{c} \sigma_{11}-\sigma_{22}=2\left(\frac{\partial W}{\partial I_{1}}+I_{1}\frac{\partial W}{\partial I_{2}}\right)\left(\lambda -\frac{1}{\lambda^{3}}\right). \end{array} \end{array}$$

#### 3.2.3. Biaxial Mode of Deformation

In this deformation case, the material is stretched unequally in two directions $\left(\sigma_{11}\neq \sigma_{22}\right)$ while the third direction is stress-free $\left(\sigma_{33}=0\right)$. The deformation gradient tensor F and the nominal stress tensor for the given deformation case are given by

(15) $$\begin{array}{c} \begin{array}{c} F=\left[\begin{array}{ccc} \lambda & 0 & 0\\ 0 & \frac{\lambda +\alpha -1}{\alpha } & 0\\ 0 & 0 & \frac{\alpha }{\lambda^{2}+\alpha \lambda -\lambda } \end{array}\right],\;\sigma =\left[\begin{array}{ccc} \sigma_{11} & 0 & 0\\ 0 & \sigma_{22} & 0\\ 0 & 0 & 0 \end{array}\right], \end{array} \end{array}$$

where α>0 is a positive constant. Using the above expressions (15) in (10), the nominal principal stresses under biaxial deformation case are obtained as

(16) $$\begin{array}{c} \begin{array}{c} \sigma_{11}=2\left(\frac{\partial W}{\partial I_{1}}+I_{1}\frac{\partial W}{\partial I_{2}}\right)\left[\lambda -\frac{\alpha^{2}}{\lambda {\left(\lambda^{2}+\alpha \lambda -\lambda \right)}^{2}}\right]-2\left(\frac{\partial W}{\partial I_{2}}\right)\left[\lambda^{3}-\frac{\alpha^{4}}{\lambda {\left(\lambda^{2}+\alpha \lambda -\lambda \right)}^{4}}\right],\\ \sigma_{22}=2\left(\frac{\partial W}{\partial I_{1}}+I_{1}\frac{\partial W}{\partial I_{2}}\right)\left[\frac{\lambda +\alpha -1}{\alpha }-\frac{\alpha^{3}}{\left(\lambda +\alpha -1\right){\left(\lambda^{2}+\alpha \lambda -\lambda \right)}^{2}}\right]\\ -2\left(\frac{\partial W}{\partial I_{2}}\right)\left[{\left(\frac{\lambda +\alpha -1}{\alpha }\right)}^{3}-\frac{\alpha^{5}}{\left(\lambda +\alpha -1\right){\left(\lambda^{2}+\alpha \lambda -\lambda \right)}^{4}}\right]. \end{array} \end{array}$$

#### 3.2.4. Equibiaxial Mode of Deformation

In this deformation case, the material is stretched equally in two directions $\left(\sigma_{11}=\sigma_{22}\right)$ while the third direction is stress-free $\left(\sigma_{33}=0\right)$. This deformation case is considered as a special case of the above biaxial deformation case for α=1. On substituting α=1 in the above Equation (16), the nominal stress for the given equibiaxial deformation case is given by

(17) $$\begin{array}{c} \begin{array}{c} \sigma_{11}=\sigma_{22}=2\left(\frac{\partial W}{\partial I_{1}}+I_{1}\frac{\partial W}{\partial I_{2}}\right)\left(\lambda -\frac{1}{\lambda^{5}}\right)-2\left(\frac{\partial W}{\partial I_{2}}\right)\left(\lambda^{3}-\frac{1}{\lambda^{9}}\right). \end{array} \end{array}$$

### 3.3. A Newly Proposed Material Model

There are so many existing material models [34] that are frequently used to model the hyperelastic behavior of an elastomeric material class. Such material models are phenomenologically defined using mathematical expressions of polynomial, exponential, and logarithmic terms [36,37]. The central aim of such early proposed material models was to accurately capture the experimental data, particularly a uniaxial test of deformation. At the same time, the standard deformation modes such as pure shear, biaxial, and biaxial tests are generally becoming ineffective in capturing the material behavior of elastomeric materials with identical values of material parameters used to fit the model to experimental data. This fact creates hurdles in connecting fitting procedures and computational calculations with physical explanations of the hyperelastic deformation of an elastomeric material class. To the best of our knowledge, no existing hyperelastic material model captures all the modes of deformations data of uniaxial, biaxial, and pure shear-based experimental tests with the same values of material parameters with a low margin of error. However, very few material models [37,51] exist that can capture experimental data of uniaxial tension, equibiaxial tension, and pure shear deformation with significant-margin of error. Furthermore, such existing material models contain many mathematical complexities that are not good for computational calculations. In this context, an alternative form of strain energy function $W\left(I_{1},I_{2}\right)=\mu \left(W_{1}+W_{2}+W_{3}\right)$ consisting of one exponential term $W_{1}$ and two logarithmic terms $W_{2}$ and $W_{3}$ is proposed as

(18) $$\begin{array}{c} \begin{array}{c} W_{1}=\frac{1}{a}\exp\left(a[I_{1}-3]\right)-\frac{1}{a},\;W_{2}=b\left(I_{1}-2\right)\left[1-\ln\left(I_{1}-2\right)\right]-b,\\ W_{3}=c\left[1-\ln\left(\frac{1}{I_{1}-2}\right)\right]-c, \end{array} \end{array}$$

where *a*, *b*, and *c* are the material parameters. All together, the exponential term $W_{1}$ and two logarithmic terms $W_{2}$ and $W_{3}$ manifest the proposed “Exp-ln-ln” strain energy density function given as

(19) $$\begin{array}{c} \begin{array}{c} W=\mu \left[\frac{1}{a}\exp\left(a\left[I_{1}-3\right]\right)-\frac{1}{a}+b\left(I_{1}-2\right)\left[1-\ln\left(I_{1}-2\right)\right]-b+c\left[1-\ln\left(\frac{1}{I_{1}-2}\right)\right]-c\right]. \end{array} \end{array}$$

Moreover, the proposed “Exp-ln-ln” type of new strain energy function (19) satisfies all the necessary and sufficient conditions to predict the mechanical behavior of the elastomeric material class as (i) it disperses in undeformed configuration (i.e., at $W_{\left(I_{1}-3\right)}=3$), (ii) it tends towards infinity at large deformations along with the corresponding stress, and (iii) it satisfies zero stress value at undeformed configuration. In the proposed strain energy function (19), the material parameter μ physically signifies the shear modulus, *a* explicitly linked with the limiting chain extensibility of monomers in polymeric chains, *b* accounts for material micro-voids, porosity, and molecular chain breakage, and *c* physically linked with the strength of intermolecular forces between chain molecules.

## 4. Results and Discussions

This section firstly validates the analytical findings of a newly proposed material model (19) in previous Section 3 with the experimental data set obtained in Section 2 for Latex, Oppo, and Ecoflex elastomers. Then, the corresponding material constants of a newly proposed material model (19) are identified for each tested material specimen of the mentioned elastomers. Later, a summarized mechanical comparison of Latex, Oppo, and Ecoflex elastomers used in morphing wing applications is also discussed, connecting with the experimentally validated analytical findings of the current study.

### 4.1. Identification of Material Parameters

**Figure 5:**
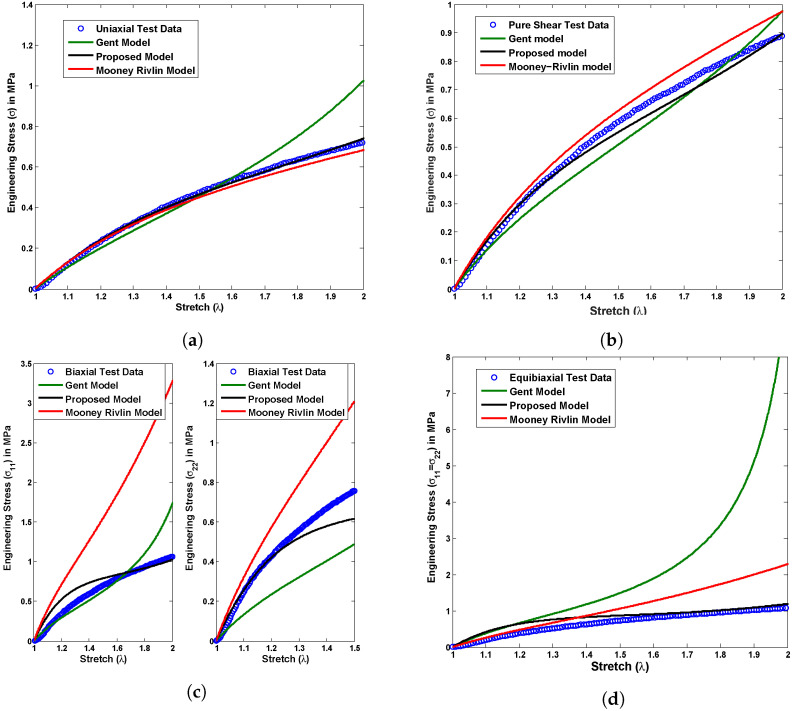
Experimental validation of the proposed model with Latex test data compared to the existing material models under all possible deformation modes, (**a**) uniaxial, (**b**) pure shear, (**c**) biaxial, and (**d**) equibiaxial for a single set of material constants.

**Figure 6:**
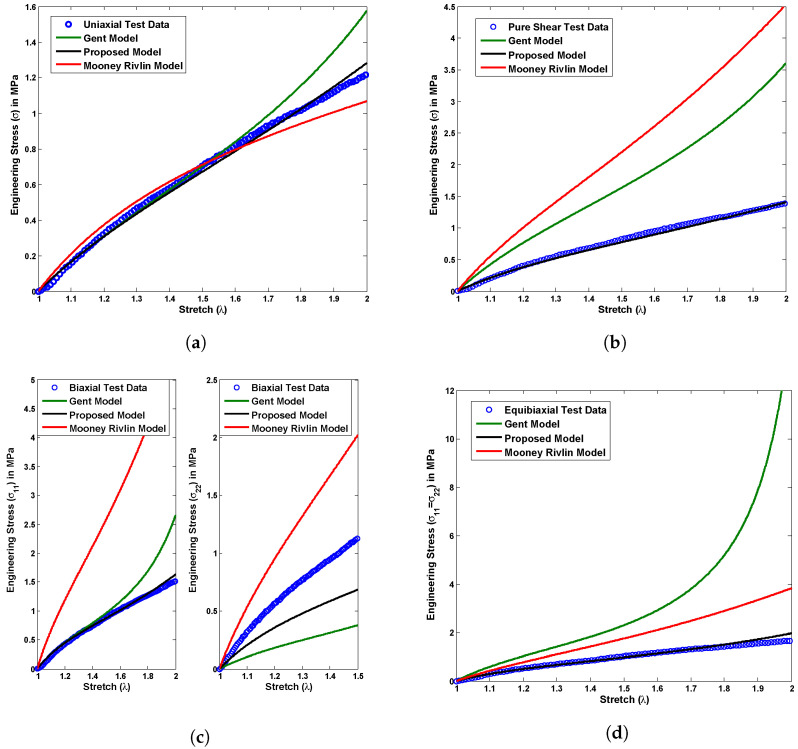
Experimental validation of the proposed model with Oppo test data compared to the existing material models under all possible deformation modes, (**a**) uniaxial, (**b**) pure shear, (**c**) biaxial, and (**d**) equibiaxial for a single set of material constants.

**Figure 7:**
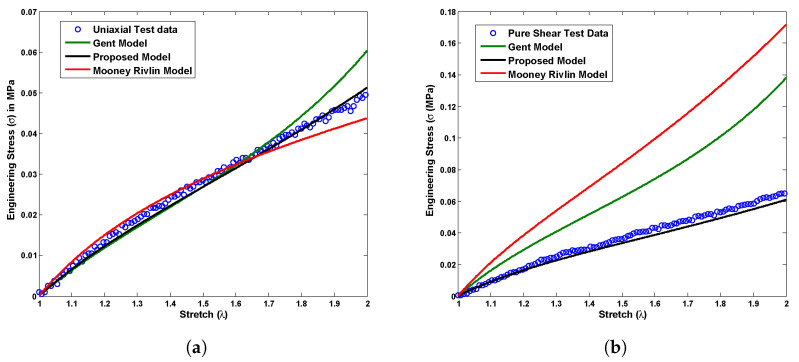

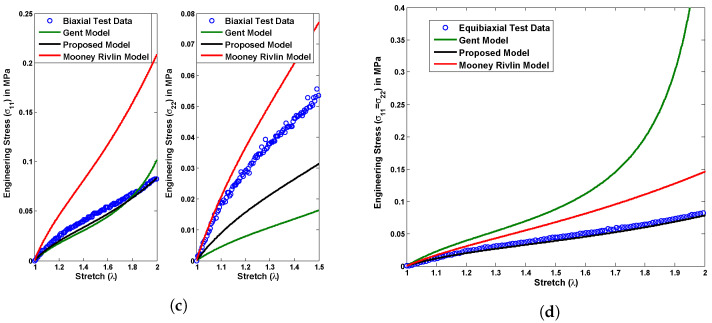
Experimental validation of the proposed model with Ecoflex test data compared to the existing material models under all possible deformation modes, (**a**) uniaxial, (**b**) pure shear, (**c**) biaxial, and (**d**) equibiaxial for a single set of material constants.

Experimental validation of any material model is commonly examined based on how accurately the model works in different regions of the stress-strain curve. In the related studies [36,37], the authors used well-known uniaxial experimental data in general by fitting a set of material constants. The authors commonly ignored checking the fitting accuracy of the material model with the same material constants in other biaxial and pure shear modes of deformations. If they do so, they may find that various material models do not accurately fit all the modes of deformations with a single set of material constants. In this regard, we perform an exercise on the experimental validity of our newly proposed material model (19). The model (19) is fitted with three different elastomers-based tested data investigated in previous Section 2 in all possible deformation modes compared with a few well-known existing material models, namely, the Mooney Rivlin and Gent given by

(20) $$\begin{array}{c} \begin{array}{c} W_{MR}=C_{1}\left(I_{1}-3\right)+C_{2}\left(I_{2}-3\right),\;W_{G}=\frac{-\mu }{2}J_{m}\ln\left(1-\frac{I_{1}-3}{J_{m}}\right), \end{array} \end{array}$$

where $C_{1}$ and $C_{2}$ are the Mooney Rivlin material constants, whereas μ and $J_{m}$ represent the Gent material parameters. A group of stress versus stretch plots shown in Figure 5, Figure 6 and Figure 7 demonstrates a material constant fitting exercise for the material models in all possible modes of deformations such as uniaxial, pure shear, biaxial, and equibiaxial for Latex, Oppo, and Ecoflex elastomers, respectively. The corresponding set of material parameters of the considered material models are indicated in Table 1, Table 2 and Table 3 for Latex, Oppo, and Ecoflex elastomers, respectively.

Figure 5 presents a comparison among currently tested Latex elastomer data under all possible deformation modes such as uniaxial, pure shear, biaxial, and equibiaxial and the above-mentioned material models in (19) and (20). The proposed material model (19) shows exact one-to-one corroboration with uniaxial test data and a qualitative agreement with another pure shear, biaxial, and equibiaxial test data for a single set of material constants calculated in Table 1. On the other hand, the Mooney Rivlin model does not show a good agreement with any type of deformation mode test data except uniaxial. At the same time, the Gent material model does not fit properly any tested data investigated here, not even for a uniaxial case. The primary reason behind such disagreements is a restriction in varying material constants while changing the deformation modes, such as uniaxial to biaxial or pure shear to equibiaxial. Figure 6 presents a comparison among currently tested Oppo elastomer data under all possible deformation modes such as uniaxial, pure shear, biaxial, and equibiaxial and above-mentioned material models in (19) and (20). Similarly, Figure 7 presents a comparison among currently tested Ecoflex elastomer data under all possible deformation modes, the proposed material model (19), Moony Rivlin model (20), and Gent material model (20). Figure 6 and Figure 7 both repeat the similar capturing trends of Figure 5 as accurately fitting the proposed material model (19) and poorly fitting the existing Moony Rivlin and Gent material models (20). The respective single set of material constants for Oppo and Ecoflex elastomers are calculated in Table 2 and Table 3, respectively.

In practice, there is a possibility that the Gent material model may accurately fit the tested data after varying a single set of material constants fitted from an uniaxial deformation case. However, in actual practical conditions, this should not be allowed. Hence, the proposed material model (19) is the only qualified material model that accurately captured the currently tested data for Latex, Oppo, and Ecoflex elastomers account for flexible skin-based aircraft morphing wing applications.

### 4.2. Mechanical Comparison of Latex, Oppo, and Ecoflex Elastomers

It is evident that the material for morphing wings cannot be wholly rigid but should be flexible enough to morph its shape during flight operations. To meet such requirements of morphing wings, the materials must be elastic and flexible enough for easy deformation with adequate strength to bear aerodynamic loads. Additionally, the materials should easily recover to its original state with no plastic deformation as excess material causes drag that reduces efficiency markedly [10]. Elastomeric materials closely fulfill all the criteria mentioned above for plausible skin materials. Natural rubber-based elastomers such as Latex and Oppo and silicon-based materials such as Ecoflex are being utilized for the purpose. Latex and Oppo have the advantage of higher fracture toughness and large elasticity with excellent durability. At a particular condition, both have higher stiffness (μ = 0.3–0.4 MPa), which accounts for a higher actuation force requirement. On the other hand, Ecoflex, which can be easily synthesized in the laboratory with different shore hardness, can be a better candidate material for the morphing wing. This is because silicone-based Ecoflex has at least ten times lower stiffness (μ = 0.012–0.02 MPa) than Oppo and Latex. Therefore, less actuation force is required to morph the wing. Moreover, earlier researchers investigated that it has lesser flaw sensitivity [52], larger elasticity [53], low hysteresis losses, and low-stress relaxations [40] that best suit its application in the morphing wings.

## 5. Concluding Remarks

The present work provides a comprehensive multiaxial experimental and theoretical study of three potential elastomers (Latex, Oppo, and Ecoflex) relevant for morphing wing application. In addition, a novel nonlinear hyperelastic constitutive model “Exp-ln-ln” with four material parameters is proposed to reasonably predict multiaxial modes of deformations (UX, PS, BX, and EB) mimicking polymorphing wing. The unique feature of the proposed model is that it reasonably fits all modes of deformations using a single set of material parameters. Limitations of the applicability of the proposed material model apply only to a few elastomers in biaxial transverse stress states ($\sigma_{22}$) with a single set of material constants fitted with uniaxial test data. This needs further refinement of the currently proposed material model in the future. Nevertheless, except for such may or may not capture transverse stresses ($\sigma_{22}$), the proposed material model qualitatively fitted all the elastomeric experimental data in the current study, eases the modeling and simulation task of morphing wing. Moreover, the major conclusion from the present work is that silicone-based skin, including Ecoflex, has superior characteristics for morphing wings (monomorphing and polymorphing) owing to its significantly less stiffness leading to lesser actuation force requirement.

## Figures and Tables

**Table 1:** Material parameters of proposed (19), Mooney Rivlin (20), and Gent (20) material models for Latex.

Constitutive Model	Material Constants
Proposed model	μ=0.30 MPa, a=0.08, b=0.28, c=0.15
Mooney Rivlin model	$C_{1}=0.14$ MPa, $C_{2}=0.11$ MPa
Gent model	μ=0.39 MPa, $J_{m}=6$

**Table 2:** Material parameters of proposed (19), Mooney Rivlin (20), and Gent (20) material models for Oppo.

Constitutive Model	Material Constants
Proposed model	μ=0.39 MPa, a=0.1, b=0.0018, c=0.001
Mooney Rivlin model	$C_{1}=0.21$ MPa, $C_{2}=0.19$ MPa
Gent model	μ=0.6 MPa, $J_{m}=6$

**Table 3:** Material parameters of proposed (19), Mooney Rivlin (20), and Gent (20) material models for Ecoflex.

Constitutive Model	Material Constants
Proposed model	μ=0.012 MPa, a=0.1, b=0.0018, c=0.001
Mooney Rivlin model	$C_{1}=0.009$ MPa, $C_{2}=0.007$ MPa
Gent model	μ=0.023 MPa, $J_{m}=6$

## Data Availability

Not applicable.
